# Understanding the full clinical spectrum of childhood diarrhoea in low-income and middle-income countries

**DOI:** 10.1016/S2214-109X(19)30056-7

**Published:** 2019-05

**Authors:** Michelle J Groome, Umesh D Parashar

**Affiliations:** South African Medical Research Council: Respiratory and Meningeal Pathogens Research Unit, University of the Witwatersrand, 2013 Johannesburg, South Africa (MJG); and Centers for Disease Control and Prevention, Atlanta, GA, USA (UDP)

Diarrhoea remains a leading cause of morbidity and mortality in young children, particularly in low-income and middle-income countries. Although childhood diarrhoea mortality globally declined by nearly 60% from 2000 to 2016, its incidence showed a relatively modest decline of about 13%.^1^ In addition to their short-term effects, diarrhoea episodes are associated with childhood growth faltering, which predisposes children to further episodes of infectious diseases.^2,3^ Improved understanding of the burden, aetiology, and long-term adverse consequences of the full clinical spectrum of childhood diarrhoea is required to optimise treatment, prevention, and control strategies.

Two recent landmark multicountry studies have vastly advanced our knowledge of the aetiology and outcomes of moderate-to-severe diarrhoea (MSD) requiring health care (the Global Enteric Multicentre Study [GEMS]^4^) and diarrheal episodes in community settings among children in low-income and middle-income countries (the Etiology, Risk Factors, and Interactions of Enteric Infections and Malnutrition and the Consequences for Child Health and Development [MAL-ED] study^5^). In their Article published in *The Lancet Global Health*, Karen Kotloff and colleagues^6^ did a 1-year extension of GEMS, called GEMS-1A, to examine the disease burden, aetiology, and adverse clinical outcomes of less-severe diarrhoea (LSD) requiring health care, thereby completing the characterisation of the full clinical spectrum of childhood diarrhoea in low-income and middle-income countries.

The overall incidence of LSD among infants was almost four times higher than that of MSD, highlighting this substantial additional burden of medically attended diarrheal disease over and above that of hospitalisations and deaths. Furthermore, children with MSD and LSD were both vulnerable to linear growth faltering in the 60 days after the diarrheal episode, indicating that prevention of diarrhoea of any severity could improve their adverse nutritional consequences.

Like in GEMS, four pathogens—rotavirus, *Cryptosporidium* spp*, Shigella* spp, and enterotoxigenic *Escherichia coli* producing heat-stable toxin—were responsible for the majority of MSD and LSD cases in GEMS-1A, and these pathogens were also frequently identified in community diarrhoea cases in the MAL-ED study.^5,6^ These are clearly important targets for pathogen-specific interventions. In GEMS-1A, norovirus GII and adenovirus 40/41 played a greater role in LSD cases than in MSD cases in children aged 0–11 months. In MAL-ED, *Campylobacter* spp, norovirus GII, and astrovirus were important causes of community diarrhoea episodes.^5^ Collectively, these data indicate an aetiological role for a wider range of enteric pathogens in milder diarrhoea cases and support the value of non-pathogen-specific measures—such as improved hygiene and nutrition—to reduce the burden of childhood diarrhoea.

Interestingly, *Helicobacter pylori* was significantly associated with diarrhoea in the GEMS-1A study and appeared in the top five ranking agents in nearly all age groups. Testing for this pathogen had not been conducted in the previous studies,^4,5^ and the significance of *H pylori* as a diarrheal pathogen in children thus requires further investigation. *H pylori* infection might also play a role in iron deficiency, growth impairment, malabsorption, and cognitive function, although studies assessing these manifestations of *H pylori* have yielded some conflicting results.^7^ Particularly in resource-poor countries, with a high prevalence of malnutrition and enteric co-infection, the role of *H pylori* and the associated hypochlorhydria remains unclear and is an area for future research.

Use of quantitative molecular diagnostic methods has improved detection of enteric pathogens resulting in improved estimates of pathogen-specific burdens of childhood diarrhoea. For example, the incidence of most pathogens in the original GEMS study were increased when quantitative quantitative PCR methods, rather than traditional methods, were used for testing.^8^ In particular, adenovirus 40/41 and *Campylobacter* spp featured in the top six most attributable pathogens after reanalysis. Similarly, reanalysis of MAL-ED samples using PCR substantially altered previous estimates of diarrhoea aetiology, with markedly increased estimates for *Shigella* spp and adenovirus 40/41, in particular.^9^ Unfortunately, quantitative molecular diagnostic methods were not used in the GEMS-1A study, so incidence of certain pathogens might have been underestimated.

Rotavirus vaccines are now being used in nearly 100 countries globally, including many low-income and middle-income countries, and continued monitoring is needed to assess possible changes in the aetiology of diarrhoea post-introduction. The Vaccine Impact on Diarrhea in Africa (VIDA) study, currently being conducted in The Gambia, Mali, and Kenya, will provide valuable data on changes in aetiological pathogens after rotavirus vaccination introduction. Yet, simpler, more cost-effective alternatives to these large-scale case-control studies are needed. Molecular diagnostic testing has recently been successfully piloted in WHO-coordinated Global Rotavirus Surveillance Network, which represents a broad range of countries.^10^ Leveraging existing networks in this way could provide a comprehensive and cost-effective complementary source of aetiological data moving forward, while also simplifying protocols for enteric testing across countries. With ongoing focused global efforts to increase the coverage of rotavirus vaccines and the promise of new and improved vaccine candidates against several enteric pathogens in the not-too-distant future, the fight to reduce diarrheal disease in our children continues unabated.
